# Nucleolar Localization of the RNA Helicase DDX21 Predicts Survival Outcomes in Gynecologic Cancers

**DOI:** 10.1158/2767-9764.CRC-24-0001

**Published:** 2024-06-13

**Authors:** Marwa W. Aljardali, Kevin M. Kremer, Jessica E. Parker, Elaine Fleming, Hao Chen, Jayanthi S. Lea, W. Lee Kraus, Cristel V. Camacho

**Affiliations:** 1 Laboratory of Signaling and Gene Regulation, Cecil H. and Ida Green Center for Reproductive Biology Sciences, University of Texas Southwestern Medical Center, Dallas, Texas.; 2 Division of Basic Research, Department of Obstetrics and Gynecology, University of Texas Southwestern Medical Center, Dallas, Texas.; 3 Division of Gynecologic Oncology, Department of Obstetrics and Gynecology, University of Texas Southwestern Medical Center, Dallas, Texas.; 4 Department of Pathology, University of Texas Southwestern Medical Center, Dallas, Texas.

## Abstract

Cancer cells with DNA repair defects (e.g., *BRCA1/2* mutant cells) are vulnerable to PARP inhibitors (PARPi) due to induction of synthetic lethality. However, recent clinical evidence has shown that PARPi can prevent the growth of some cancers irrespective of their *BRCA1/2* status, suggesting alternative mechanisms of action. We previously discovered one such mechanism in breast cancer involving DDX21, an RNA helicase that localizes to the nucleoli of cells and is a target of PARP1. We have now extended this observation in endometrial and ovarian cancers and provided links to patient outcomes. When PARP1-mediated ADPRylation of DDX21 is inhibited by niraparib, DDX21 is mislocalized to the nucleoplasm resulting in decreased rDNA transcription, which leads to a reduction in ribosome biogenesis, protein translation, and ultimately endometrial and ovarian cancer cell growth. High PARP1 expression was associated with high nucleolar localization of DDX21 in both cancers. High nucleolar DDX21 negatively correlated with calculated IC_50_s for niraparib. By studying endometrial cancer patient samples, we were able to show that high DDX21 nucleolar localization was significantly associated with decreased survival. Our study suggests that the use of PARPi as a cancer therapeutic can be expanded to further types of cancers and that DDX21 localization can potentially be used as a prognostic factor and as a biomarker for response to PARPi.

**Significance:**

Currently, there are no reliable biomarkers for response to PARPi outside of homologous recombination deficiency. Herein we present a unique potential biomarker, with clear functional understanding of the molecular mechanism by which DDX21 nucleolar localization can predict response to PARPi.

## Introduction

Ovarian cancer is the fifth leading factor in women's cancer-related fatalities (1). Most patients are diagnosed at an advanced stage with disseminated disease (2, 3). No less than 15% of patients with high-grade serous ovarian cancer have an inherited predisposition which is predominantly caused by *BRCA1/2* mutations (3, 4). These mutations have served as genetic biomarkers that have led to therapeutic application (5). Recently, PARP1 inhibitors have garnered significant attention in the treatment of *BRCA1/2* mutant tumors because it was demonstrated that PARP inhibition promotes synthetic lethality in *BRCA1/2* mutant cancers which are deficient in homologous recombination (HR) repair of damaged DNA (6, 7). Over the last decade, PARP inhibitors (PARPi) olaparib, niraparib, and rucaparib have been introduced to the standard care of platinum-sensitive ovarian cancer after recurrence as maintenance and as treatment (2, 8). Also, since 2020 PARPi have been approved to be used for maintenance of newly diagnosed advanced ovarian cancer with *BRCA1/2* mutation (2, 8). Thus, for patients with *BRCA1/2* mutations, PARPi have transformed ovarian cancer patient care by providing a considerable clinical benefit (4, 9, 10).

For advanced stage recurrent endometrial cancer, survival is poor and treatment options are limited, reflecting an unmet need in gynecologic cancer care (11). Recent studies including a phase III clinical trial have suggested that combining PARPi with immunotherapeutic agents in endometrial cancers can have a significant clinical efficacy (12–14). Clinical trials are currently assessing the role of different PARPi as monotherapy and in combination with immune checkpoint inhibitors in various settings of endometrial cancer focusing on DNA damage–related mechanisms of action (12). Although PARPi are not yet FDA approved for endometrial cancer, a recent case report presented a patient with recurrent endometroid endometrial cancer with HR-related mutation (*BRIP1*) who had a complete pathologic response to olaparib indicating a promising effect for PARPi in endometrial cancer (15).

PARP1 is a ubiquitously expressed nuclear enzyme that uses nicotinamide adenine dinucleotide (NAD^+^) to catalyze the addition of poly(ADP-ribose; PAR) polymers on substrate proteins (ADPRylation; refs. 16–18). Although PARP1 is essential for many biological processes, historically studies of PARP1 have focused heavily on its role in DNA damage repair and genome maintenance (18–21). However, the biology of PARP1 is much broader than that (22–24). Interestingly, several studies have now shown efficacy irrespective of *BRCA1/2* status for olaparib, rucaparib, and niraparib in recurrent/relapsed ovarian cancer (10, 25–32). Although PARPi monotherapy is no longer recommended for chemosensitive, nonmutated *BRCA1/2* cancers, data on the use of PARPi in this setting are still making progress so the decision to provide PARPi should be based on an individualized analysis (33). These studies suggest that DNA repair is not the only basis for PARPi-positive therapeutic action and indicate that there is a broader utility for PARPi that is currently not being exploited.

Uncovering how PARPi function in a manner independent of DNA damage exposes new vulnerabilities in cancer that may allow the expansion of their use as cancer therapeutics. Interestingly, we recently described one such mechanism in breast cancer, wherein PARP1-mediated ADPRylation of the DEAD-box nucleolar RNA helicase DDX21 is required for its proper localization and retention to the nucleolus. Treatment of breast cancer cells with PARPi resulted in the mislocalization of DDX21 to the nucleoplasm, leading to diminished ribosome biogenesis, protein translation, and cell proliferation (19). DDX21 has been shown to bind rDNA chromatin and plays a role in several steps of the ribosome biogenesis process which is initiated in the nucleolus (34). In the absence of cellular stress, about 40% of PARP1 protein stays in the nucleolus and participates in ribosome biogenesis (35), in part through ADPRylation-mediated regulation of DDX21 (19).

Herein, we expand our mechanistic observation in other cancer types and show an association between DDX21 nucleolar localization and clinical outcome. Our cell-based assays using endometrial and ovarian cancer cell lines further validate a novel PARPi mechanism of action where PARPi disrupts the nucleolar localization of DDX21 leading to decreased ribosome biogenesis, protein translation, and ultimately, inhibition of cell proliferation. Analysis of our endometrial cancer patient cohort indicates the potential of DDX21 nucleolar localization to be used as a novel prognostic biomarker to predict clinical outcome.

## Materials and Methods

### Cell Culture

OVCAR3 and OVCAR4 serous ovarian cancer cell lines were purchased from the ATCC (OVCAR3: ATCC, RRID: CVCL_0465; OVCAR4: ATCC, RRID: CVCL_1627) and were cultured as recommended. Patient-derived high-grade serous ovarian cancer cell line HCC5012 was obtained directly from source (Dr. Adi Gazdar). Cells were cultured in RPMI 1640 media supplemented with 10% FBS, 2 mmol/L l-glutamine, and 1% penicillin/streptomycin. Endometroid endometrial carcinoma cell lines HEC-1-A and KLE were purchased from ATCC (HEC-1-A: ATCC; catalog no. HTB-112; RRID: CVCL_0293, KLE: ATCC; catalog no. CRL-1622; RRID: CVCL_1329) and Ishikawa was purchased from Sigma-Aldrich (catalog no. 99040201; RRID: CVCL_2529). HEC-1-A cell line was maintained in McCoy's 5A (ATCC) supplemented with 10% FBS and 1% penicillin/streptomycin. The KLE cell line was maintained in DMEM (ATCC) supplemented with 10% FBS and 1% penicillin/streptomycin. The Ishikawa cell line was maintained in minimum essential medium with glutamine (Thermo Fisher Scientific) supplemented with 5% FBS, and 1% non-essential amino acids. All cell lines were authenticated for cell type identity using the GenePrint 24 system (Promega, B1870), and confirmed as *Mycoplasma*-free every 6 months using the Universal Mycoplasma Detection Kit (ATCC, 30-1012K). Fresh cell stocks were regularly replenished from the original stocks every few months (no more than 15 passages).

### Antibodies

The custom rabbit polyclonal antiserum against PARP1 used for immunofluorescent staining assays was generated by using an antigen comprising the amino-terminal half of PARP1 (ref. 36; now available from Active Motif, 39559; RRID: AB_2793257). The custom recombinant antibody-like anti-ADP-ribose binding reagent (PAR) was generated and purified in-house (ref. 37; now available from EMD Millipore, MABE1031; RRID: AB_2665467). The other antibodies used for IHC were as follows: DDX21 rabbit polyclonal antibody (Proteintech, 10528-1-AP; RRID: AB_2092705), NOP58 rabbit polyclonal antibody (Invitrogen, PA5-5432; RRID: AB_2644745), Puromycin mouse mAb (Millipore, MABE343; RRID: AB_2566826), RAD51 (Millipore, ABE257; RRID: AB_10850319), Geminin (F-7; Santa Cruz Biotechnology, sc74456; RRID: AB_1124963), and β-tubulin rabbit polyclonal antibody (Abcam, ab6046; RRID: AB_2210370).

### Immunofluorescent Staining and Confocal Microscopy

OVCAR3, OVCAR4, HCC5012, Ishikawa, HEC-1-A, and KLE cells were seeded at 4 × 10^4^ cells per well on 4-well chambered slides (Thermo Fisher Scientific, 154534) one day prior to treatment. The following day, cells were treated with niraparib (MedChem Express, HY10619) 20 µmol/L for 2 hours (for the DDX21 staining) or 10 µmol/L for 24 hours (for the RAD51 assay). The cells were washed two times with PBS, fixed in 4% paraformaldehyde for 15 minutes at room temperature, and washed three times with PBS. The cells were permeabilized for 5 minutes using 0.5% Triton X-100 in PBS, washed three times with PBS, and incubated for 20 minutes at room temperature in Blocking solution (5% BSA in PBS), and then washed one time with PBS. The fixed cells were incubated at 4°C with antibody against NOP58 or DDX21 (for the DDX21/NOP58 staining) or with antibodies against Geminin and RAD51 (for the geminin/RAD51 co-stain) in PBS overnight. The cells were then washed three times with PBS and then incubated with Alexa Fluor 488 goat anti-rabbit IgG (Thermo Fisher Scientific, A-11008; RRID: AB_143165) or Alexa Fluor 568 goat anti-mouse (Thermo Fisher Scientific, A-11031; RRID: AB_144696) in PBS for 1 hour. After incubation, the cells were then washed three times with PBS. Finally, the slides were treated with VectaShield with DAPI (Vector Laboratories, H-1200; RRID: AB_2336790) and images were acquired using an inverted Zeiss LSM 780 confocal microscope.

### Luciferase Activity Assay

Cells were seeded in opaque-walled 96-well plate. On the next day, cells were subjected to either DMSO or 20 µmol/L niraparib for 2 hours. Then cells were lysed with lysis buffer (Promega, G7570) based on the manufacturer's protocol. Luminescent readings were obtained by the luminometer and media background was subtracted and normalized for the cell viability by the CellTiter-Glo ATP measurement system (Promega) based on the manufacturer's protocol.

### Preparation of Whole-cell Lysates and Western Blotting

The cells were collected and resuspended in Lysis Buffer (50 mmol/L Tris-HCl pH 7.5, 0.5 mol/L NaCl, 1 mmol/L ethylenediaminetetraacetic acid [EDTA], 1% NP-40, 10% Glycerol) containing 1 mmol/L DTT, 1x complete protease inhibitor cocktail (Roche, 11697498001), 250 nmol/L ADP-HPD, and 10 µmol/L PJ-34. The cells were incubated on ice for 30 minutes with occasional vortexing and then centrifuged for 15 minutes at 4°C to remove the cell debris. The supernatants were collected, aliquoted, flash frozen in liquid nitrogen, and stored at −80°C until used.

Protein concentrations for the whole-cell extracts were determined using a Bradford protein assay (Bio-Rad). For each fraction collected from the indicated conditions, 10 to 20 µg of protein were separated on an 8% to 12% polyacrylamide-SDS gel and transferred to nitrocellulose membrane. The membranes were blocked for 1 hour at room temperature in TBS with 0.05% Tween (TBS-T) containing 3% nonfat dry milk. Primary antibodies [PARP1 (1:5,000), DDX21 (1:2,000), WWE-Fc (PAR; 1:1,000), β-tubulin (1:1,000), or Puromycin (1:2,000)] were diluted in 1% nonfat dry milk and incubated with membranes for 1 hour at room temperature with gentle mixing. After extensive washing with TBS-T, the membranes were incubated with an appropriate horseradish peroxidase (HRP)-conjugated secondary antibody (Pierce) diluted in TBS-T with 1% nonfat dry milk for 1 hour at room temperature. The membranes were washed extensively with TBS-T before chemiluminescent detection using SuperSignal West Pico substrate (Thermo Fisher Scientific) and ChemiDoc system (Bio-Rad).

### qPCR

cDNA pools were prepared by extraction of nuclear RNA from cells treated with niraparib using the Qiagen RNeasy Plus Mini Kit, followed by reverse transcription using MMLV reverse transcriptase (Progmega, M150B) with random hexamer primers (Sigma-Aldrich) to generate a cDNA pool. The cDNA was analyzed by qPCR using the primer sets listed below and a LightCycler 480 real-time PCR thermocycler (Roche) for 45 cycles.

### qPCR Primers

18S rRNA forward: 5′-ACCCGTTGAACCCCATTCGTGA-3′
18S rRNA reverse: 5′-GCCTCACTAAACCATCCAATCGG-3′
28S rRNA forward: 5′-CCGTGCCTTGGAAAGCGTCGC-3′
28S rRNA reverse: 5′-CAGAGGCTGTTCACCTTGGAGA-3′
47S pre-rRNA forward: 5′-GAACGGTGGTGTGTCGTT-3′
47S pre-rRNA reverse: 5′-GCGTCTCGTCTCGTCTCACT-3′
GAPDH forward: 5′-CCACTCCTCCACCTTTGAC-3′
GAPDH reverse: 5′-ACCCTGTTGCTGTAGCCA-3′

### Monitoring Translation by Puromycin Incorporation

Translation monitoring was performed as described previously (38). Briefly, OVCAR3, OVCAR4, HCC5012, Ishikawa, HEC-1-A, and KLE cells treated with or without niraparib (20 µmol/L for 2 hours) were labeled with puromycin (10 µg/mL; Sigma, P8833) for 15 minutes. Whole-cell extracts were prepared from same number of cells in each condition. Puromycin incorporation was visualized by Western blotting with an antibody against puromycin.

### Dose Response and Cell Proliferation Assays

For the dose–response assay, OVCAR3, OVCAR4, Ishikawa, and HEC-1-A were seeded at density of 10^4^ cells per well in 12-well plates. Increasing doses of niraparib were applied, media with fresh drug was replenished every 2 days. Plates were collected and fixed at day 6. Cell proliferation was assessed using a crystal violet staining assay. OVCAR3, OVCAR4, Ishikawa, and HEC-1-A cells were plated at a density of 10^4^ cells per well in 12-well plates. The cells were grown to approximately 50% confluence (∼1 day of growth) and then treated with niraparib. The cells were collected every 2 days after treatment. After collections, the cells were washed with PBS, fixed for 10 minutes with 4% paraformaldehyde at room temperature, and stored in 4°C until all timepoints had been collected. The fixed cells were stained with 0.1% crystal violet in 20% methanol solution containing 200 mmol/L phosphoric acid. After washing to remove unincorporated stain, the crystal violet was extracted using 10% glacial acetic acid and the absorbance was read at 595 nm. All growth assays were performed a minimum of three times using independent plating of cells to ensure reproducibility.

### Commercial Tissue Microarrays

Commercial cancer microarrays were ordered from Tissuearray.com catalog (OV2001a, EM1021a, HPanA150CS03, HProA100PG03). The ovarian (OV2001a) microarray contained 133 cases of serous carcinoma, 34 mucinous adenocarcinoma, 3 adenocarcinoma, 7 endometrioid adenocarcinoma, 15 metastatic carcinoma, and 8 adjacent normal ovary tissue cores. The endometrial (EM1021a) microarray contained 96 cases of endometrioid adenocarcinoma, 1 adenosquamous carcinoma, and 5 benign endometrial cores. The pancreatic (HPanA150CS03) microarray contained 80 cases of carcinoma cores and matched normal adjacent tissue. The prostate (HProA100PG03) microarray contained 95 cases of adenocarcinoma, with 2 normal adjacent tissues, and 2 normal prostate tissue cores. IHC staining was performed on the tissue microarrays as described previously.

### IHC

Unstained, formalin-fixed, paraffin-embedded (FFPE) slides, from the initial surgery of 39 patients with endometrioid endometrial cancer were obtained from the pathology departments of the University of Texas Southwestern Medical Center and Parkland Memorial Hospital. Staining was performed on a Dako Autostainer Link 48 system. The slides are baked for 20 minutes at 60°C in a heating and drying oven. Slides are then deparaffinized using Leica Autostainer XL by washing the slides successively in five wash baths: xylene (three times), 100% ethanol (twice), and 95% ethanol (twice). The slides are hydrated using Leica AutoStainer XL by soaking the slides in deionized water. We then prepare the 1x working solution of EnVision FLEX Target Retrieval Solution and set the PT Link to preheat the solution to 74°C. Slides are immersed into the preheated EnVision FLEX Target Retrieval Solution in PT Link tanks and incubate for 20 minutes at 97°C. They are then left in the tank to cool to 70°C. Once they reach this temperature, they are removed from the PT Link tank and immediately placed in a tank with 1x room temperature EnVision FLEX Wash Buffer for 5 minutes. Following this, the slides are placed on the Autostainer Link instrument to proceed with staining. The slides are incubated with EnVision FLEX Peroxidase-Blocking Reagent (300 mL/slide) for 5 minutes, then washed once with wash buffer. We then incubate the slides with the primary antibodies for 35 minutes and then wash twice with wash buffer. The optimal staining condition for anti-PARP1 was 1:1,000 dilution with low pH, and anti-DDX21 was 1:100 with low pH. Following this, we stain with EnVision FLEX/HRP for 20 minutes (300 mL/slide) and wash four times with wash buffer. Next, EnVision FLEX DAB+ Chromogen is applied for 5 minutes (600 mL/slide) and washed twice with wash buffer. Counterstain with EnVision FLEX Hematoxylin is then performed for 5 minutes (300 mL/slide) and washed twice with wash buffer. The slides are dehydrated by quickly dipping them successively in five wash baths in the following order: 95% ethanol (20 times), 100% ethanol (20 times), 100% ethanol (20 times), xylene (20 times), xylene (20 times), xylene (20 times). Finally, two drops of mounting medium are placed on the slides and the stained tissues with a glass coverslip.

IHC staining was interpreted by a single, blinded, gynecologic pathologist. Staining for the IHC antibody was graded on a 4-point scale for low (0–1) or high (2–3) staining.

### Patient Tissue Sample Collection

Tissue samples were collected intraoperatively at the time of the patient's initial surgery under a tissue repository protocol, approved by the Institutional Review Board at the University of Texas Southwestern Medical Center (#STU-012015-019). Samples were immediately fixed in 10% neutral buffered formalin and processed for paraffin embedding (FFPE). The tissue repository was queried for patients with advanced endometrial cancer who received upfront surgery. Advanced endometrial cancer was defined as stage III or stage IV disease. Thirty-nine patients with endometrial cancer (female) with endometrioid histology were identified. Patient data were obtained from electronic medical records and included: age, race, stage, histology, chemotherapy regimens, and survival outcomes. These studies were conducted in accordance with recognized U.S. and international ethical guidelines, and written informed consent was obtained from the patients. This study was approved by the Institutional Review Board at the University of Texas Southwestern Medical Center (#STU-2018-0288).

### Data Availability Statement

The data generated in this study are available upon request from the corresponding author.

## Results

### Niraparib Disrupts the Nucleolar Localization of DDX21 in Endometrial and Ovarian Cancer Cell Lines

**FIGURE 1:**
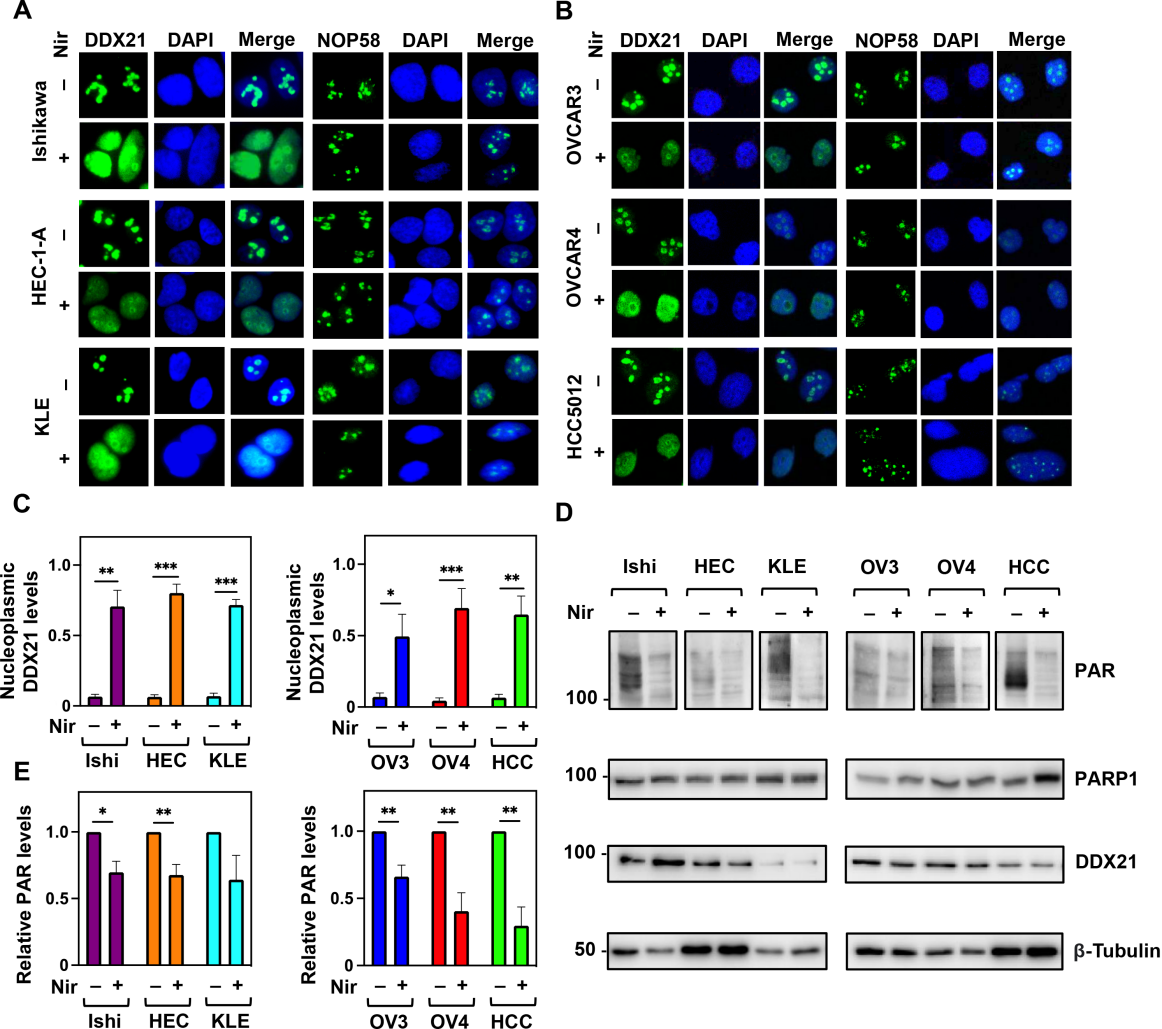
Niraparib inhibits ADPRylation and disrupts the nucleolar localization of DDX21 in endometrial and ovarian cancer cells. Inhibition of PARP1 catalytic activity in endometrial cancer cell lines (Ishikawa, HEC-1-A, KLE; **A**) and ovarian cancer cell lines (OVCAR3, OVCAR4, HCC5012; **B**) by niraparib promotes the redistribution of DDX21 from the nucleolus to the nucleoplasm but does not affect the nucleolar localization of NOP58 as assayed by immunofluorescent staining. **C,** Quantification of the results from experiments shown in A and B showing the proportion of cells that have disrupted nucleoplasmic localization of DDX21. Each bar represents the mean + SEM; *n* = 3 (endometrial), *n* = 6 (ovarian). Bars marked with asterisks are significantly different; Student *t* test; *, *P* < 0.05; **, *P* < 0.01; ***, *P* < 0.001. **D,** ADPRylation (PAR) is inhibited in endometrial and ovarian cancer cells treated with niraparib. PARP1 and DDX21 protein expression levels are not affected by niraparib treatment as assayed by Western blotting. **E,** Quantification of PAR levels from experiments shown in D for endometrial and ovarian cancer cell lines with and without niraparib treatment. Each bar represents the mean + SEM; *n* = 3. Bars marked with asterisks are significantly different; Student *t* test; *, *P* < 0.05; **, *P* < 0.01.

Our previously published work in breast cancer demonstrated that PARPi treatment of cells caused a redistribution of DDX21 from the nucleolus to the nucleoplasm (19). Thus, we sought to investigate the impact of PARPi on the localization of DDX21 in endometrial and ovarian cancer cells. We used three endometroid endometrial cancer cell lines (Ishikawa, HEC-1-A, KLE) and three ovarian cancer cell lines (OVCAR3, OVCAR4, HCC5012). On the basis of published studies (Supplementary Table S1), and the ability of all cell lines to efficiently form RAD51 foci (Supplementary Fig. S1A–S1D), all cell lines are considered HR proficient. First, we performed immunofluorescent staining of DDX21 in cells treated with and without niraparib. We quantified the number of cells displaying mislocalized nucleoplasmic DDX21. We found that niraparib indeed disrupted the nucleolar localization of DDX21, which assumed a pan-nuclear distribution after the treatment (Fig. 1A–C). However, the localization of NOP58, a nucleolar marker, did not change indicating that the nucleolar integrity was not affected by the treatment (Fig. 1A and B). Second, we evaluated the levels of ADPRylation (PAR), PARP1, and DDX21 expression in the endometrial and ovarian cancer cell lines in the presence and absence of niraparib by Western blot analysis (Fig. 1D). We showed that DDX21 and PARP1 levels remain unaffected when treated with niraparib. However, niraparib treatment caused a significant reduction in ADPRylation (Fig. 1D and E). Cell viability at this dose of niraparib (20 µmol/L for 2 hours) was not affected (Supplementary Fig. S2A). Our data indicate that niraparib inhibits ADPRylation in endometrial and ovarian cancer cell lines and is responsible for the disruption of the nucleolar localization of DDX21.

### PARP1 Inhibition Prevents Ribosome Biogenesis and Decreases Protein Translation

**FIGURE 2:**
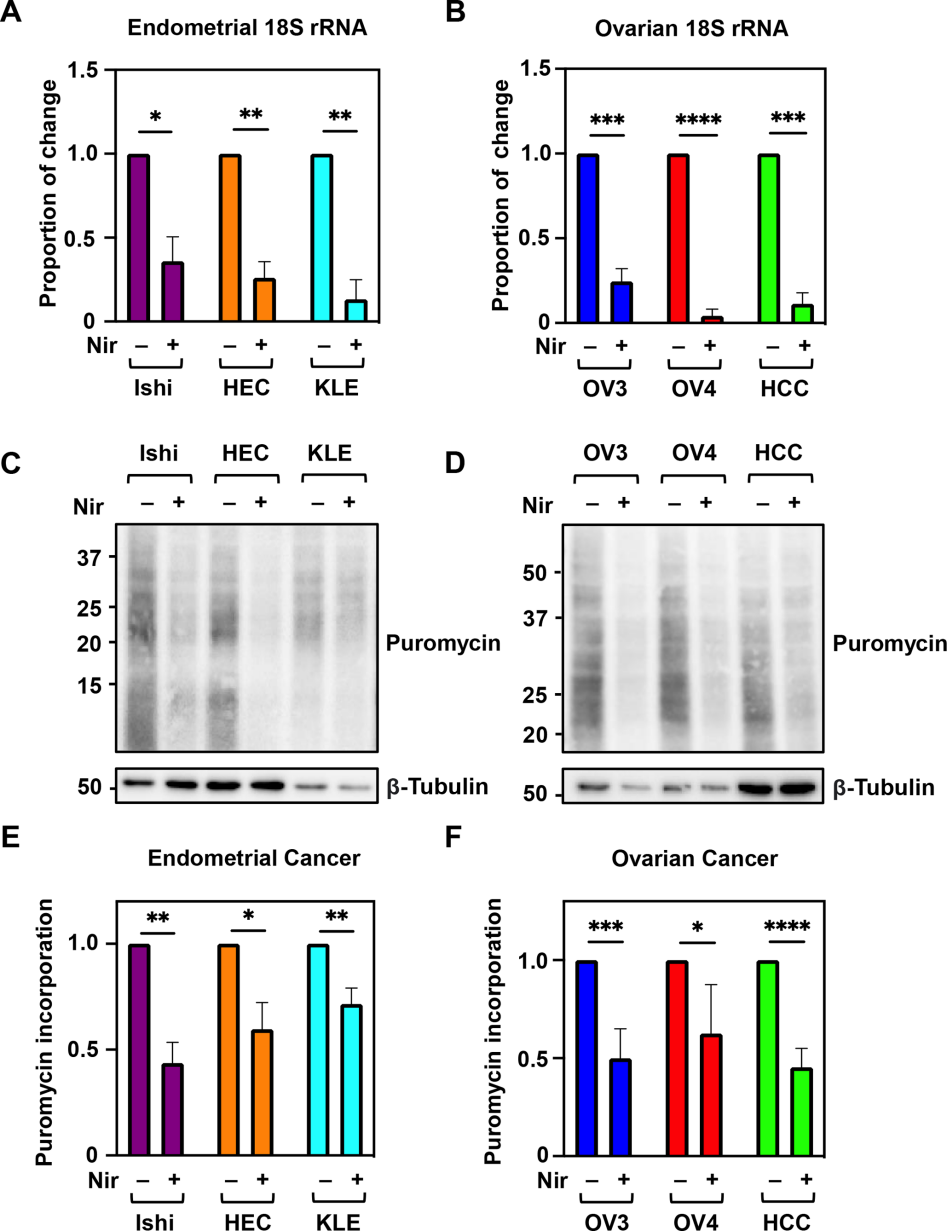
PARP1 inhibition prevents ribosome biogenesis and decreases protein translation. Treatment with niraparib significantly decreases rRNA subunit (18S) production in endometrial (**A**) and ovarian (**B**) cancer cells as assayed by qPCR. Each bar represents the mean + SEM; *n* = 3. Bars marked with asterisks are significantly different; Student *t* test; *, *P* < 0.05; **, *P* < 0.01; ***, *P* < 0.001; ****, *P* < 0.0001. Puromycin incorporation is diminished with niraparib treatment in endometrial (**C**) and ovarian (**D**) cancer cells as assayed by Western blotting. **E** and **F,** Quantification of puromycin incorporation assay from C and D, respectively. Each bar represents the mean + SEM; *n* = 3. Bars marked with asterisks are significantly different; Student *t* test; *, *P* < 0.05; **, *P* < 0.01; ***, *P* < 0.001; ****, *P* < 0.0001.

Being localized to the nucleolus, it is known that DDX21 binds the Pol I transcription complex at rDNA loci, and plays an important role in rDNA transcription and ribosome biogenesis (34). Thus, we investigated the effect of DDX21 ADPRylation on the production of rRNA. First, we measured rRNA levels by qPCR using primers designed for the rRNA subunits 18S and 28S as well as the pre-rRNA subunit 47S. Strikingly, after administration of niraparib, all endometrial and ovarian cancer cell lines showed a significant decrease in the production of rRNA subunits (Fig. 2A and B; Supplementary Fig. S2B and S2C). Second, we hypothesized that decreased levels of rRNA subunits should affect the biogenesis of ribosomes and the level of protein translation (both reduced). To test this hypothesis, we performed a puromycin incorporation assay which helped us determine the effect of DDX21 ADPRylation on protein synthesis. As expected, treatment with niraparib resulted in a significant decrease in puromycin incorporation (translation) shown by Western blot analysis (Fig. 2C–F). Collectively, our results indicate that PARP1 inhibition and reduced ADPRylation of DDX21 in endometrial and ovarian cancers decreases rDNA transcription, negatively impacting ribosome biogenesis and protein translation.

### The Nucleolar Localization of DDX21 Correlates with Sensitivity to PARPi and is Crucial for Endometrial and Ovarian Cancer Cell Growth

**FIGURE 3:**
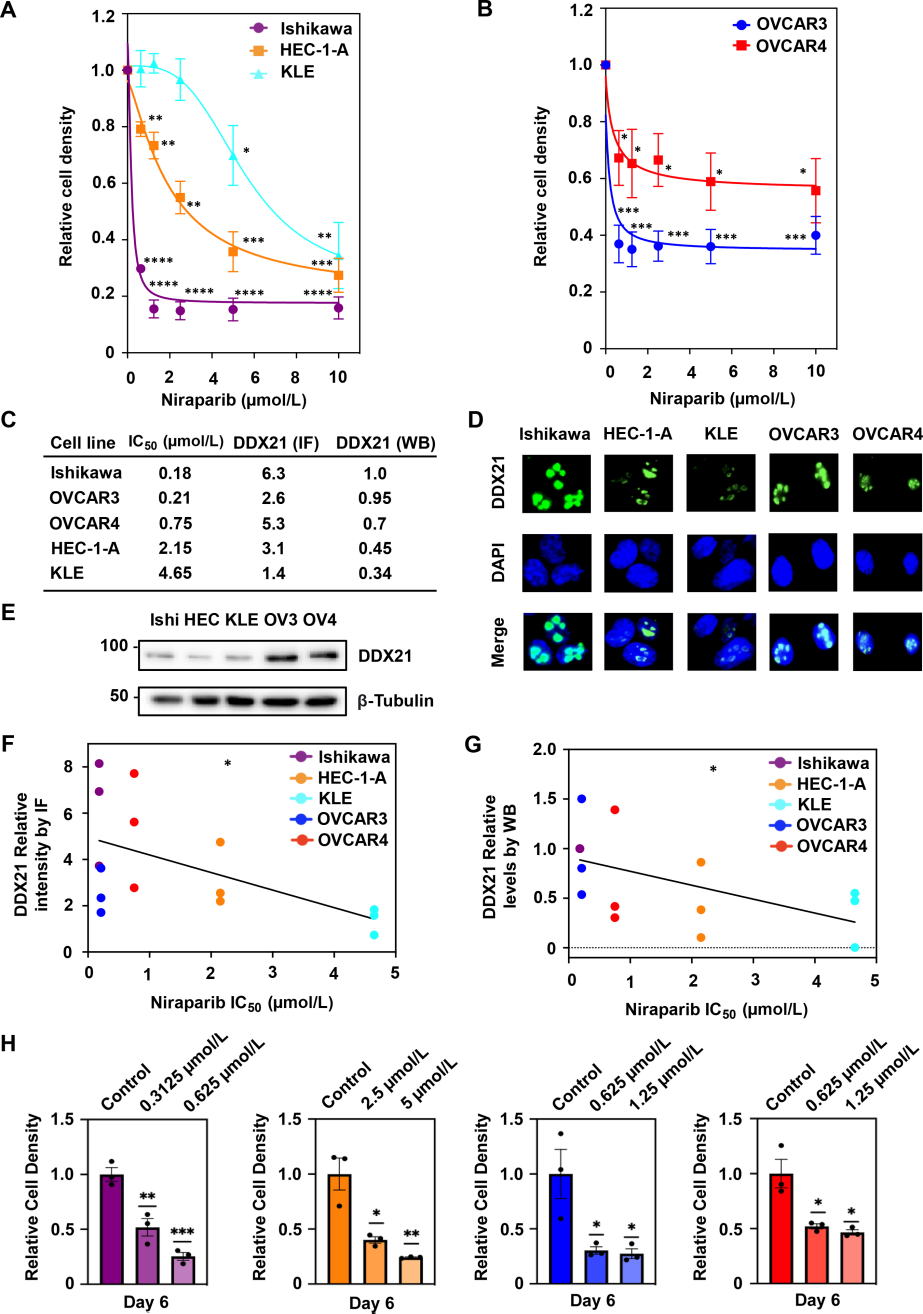
Nucleolar DDX21 predicts PARPi sensitivity and niraparib decreases cell growth in endometrial and ovarian cancers. **A** and **B,** Dose–response curves showing increasing concentrations of niraparib significantly decreasing cell viability in endometrial (A) and ovarian cancer cells (B) assayed by crystal violet staining. **C,** Table showing the IC_50_ values of niraparib in Ishikawa, HEC-1-A, KLE, OVCAR3, and OVCAR4 cells as calculated from experiments in A and B. Table also shows values for the relative levels of DDX21 as measured by immunofluorescence (IF) and Western blot (WB). Basal levels of DDX21, without any treatment in ovarian and endometrial cancer cell lines examined by immunofluorescence (**D**) and Western blotting (**E**). Correlation plots showing DDX21 intensity (*y*-axis) as measured by IF (**F**) and Western blot (**G**), and calculated IC_50_ of niraparib (*x*-axis). *n* = 3 (per cell line). Each point corresponds to a different replicate and different cell lines are colored differently; Correlation analysis; *, *P* < 0.05. **H,** PARP1 inhibition with niraparib significantly decreases the proliferation of endometrial and ovarian cancer cell lines. Bar graphs showing quantification of the growth of cells at day 6 with different concentrations of niraparib. Each bar represents the mean ± SEM; *n* = 3. Bars marked with asterisks are significantly different; Student *t* test; *, *P* < 0.05; **, *P* < 0.01; ***, *P* < 0.001.

Finally, due to the inhibition of ribosome biogenesis and protein translation through decreased nucleolar localization of DDX21, we sought to determine whether this effect would inhibit endometrial and ovarian cancer cell growth. A dose–response relationship, where we treated the endometrial cancer cells (Ishikawa, HEC-1-A, and KLE) and ovarian cancer cells (OVCAR3 and OVCAR4) with increasing doses of niraparib, showed a respective increase in cell death (Fig. 3A and B). Niraparib IC_50_ was determined for each of the five cell lines (Fig. 3C), First, we used the calculated IC_50_s to correlate to endogenous levels of nucleolar DDX21 based on immunofluorescent and Western blot intensity (Fig. 3D and E). Strikingly, we found a significant negative correlation, where high nucleolar DDX21 correlates with lower IC_50_ (Fig. 3F and G). Second, we used calculated IC_50_s to estimate two doses to be tested for the cell proliferation assays. By measuring cell proliferation over time, it was validated that niraparib causes a decrease in the proliferation of endometrial and ovarian cancer cells and the effect is most evident at day 6 (Fig. 3H; Supplementary Fig. S3A–S3C). These results bolster our hypothesis that high nucleolar DDX21 can predict response to PARPi, given that those cell lines are the most sensitive to niraparib. Altogether, our data indicate that PARP1 activity is required to retain the nucleolar localization of DDX21, which is required for the growth of endometrial and ovarian cancers and can potentially be targeted for cancer therapy.

### High Level of Nucleolar DDX21 is Associated with Decreased Survival

**FIGURE 4:**
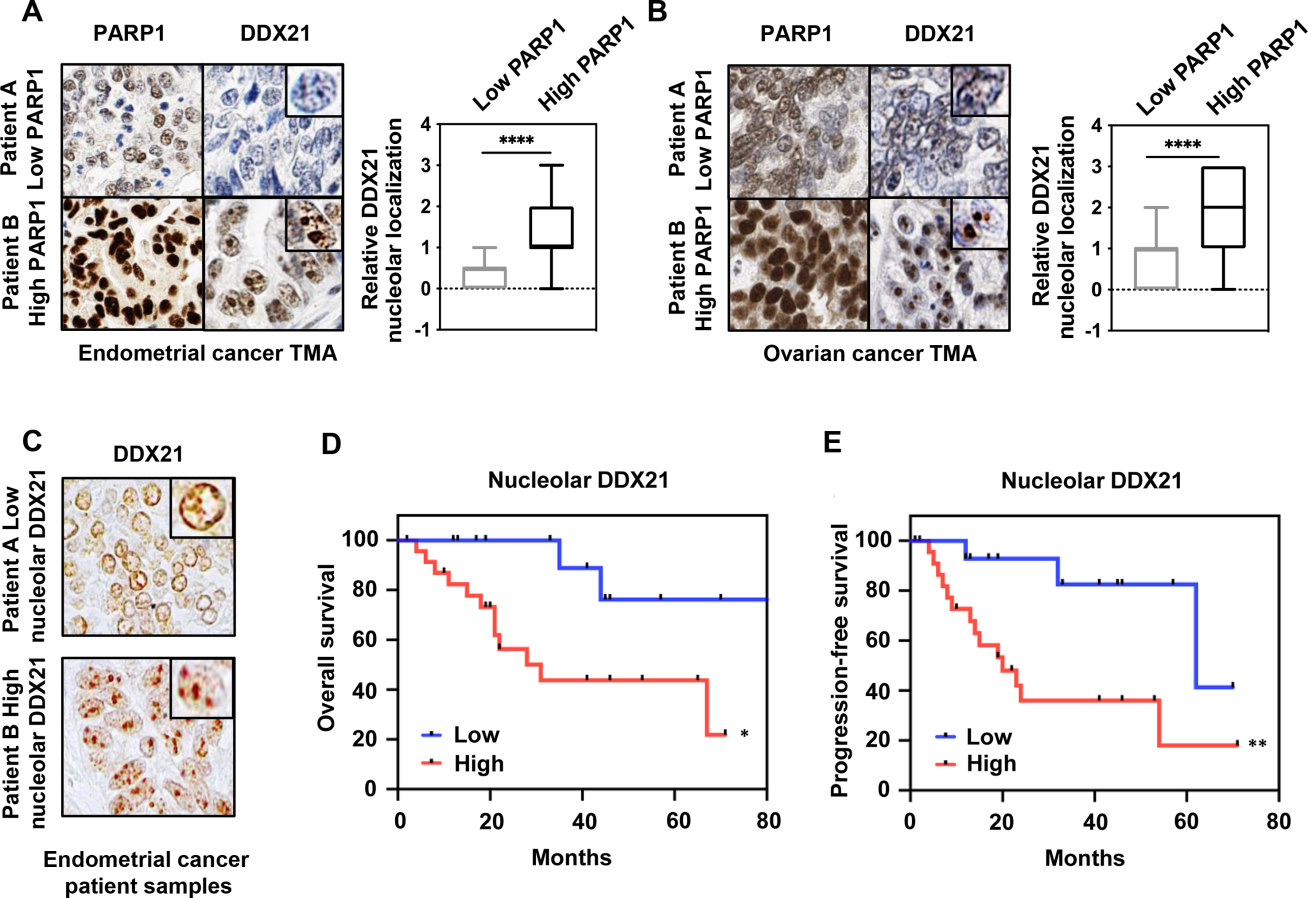
DDX21 nucleolar localization associates with PARP1 expression and decreased survival outcomes. **A** and **B,** Representative immunohistochemical staining of PARP1 and DDX21 from endometrial (A) and ovarian (B) cancer commercial tissue microarrays (left) and box plots showing quantification of nucleolar DDX21 intensity in low versus high PARP1 endometrial (A) and ovarian (B) cancer samples (right). Student *t* test; ****, *P* < 0.0001. B, Representative IHC images from advanced stage endometrioid endometrial cancer patient samples showing low versus high nucleolar DDX21 staining by IHC. Kaplan–Meier curves showing high nucleolar localization of DDX21 is significantly associated with decreased OS (*P*-value = 0.0155; **D**) and PFS (*P*-value = 0.0092; **E**) in patients with advanced stage endometrioid endometrial cancer. Total *n* = 39 (number of patients with low DDX21 nucleolar staining = 14; number of patients with high DDX21 nucleolar staining = 25). log-rank (Mantel–Cox) test; *, *P* < 0.05; **, *P* < 0.01.

In breast cancer, it was shown that high levels of PARP1 expression are associated with increased DDX21 nucleolar localization (19). We were interested in verifying this relationship in other types of cancer including endometrial, ovarian, pancreatic, and prostate cancers. We observed that both PARP1 and DDX21 are found to be more highly expressed in tumor tissues compared with normal tissues for these four cancer types [The Cancer Genome Atlas (TCGA) and GTEx; Supplementary Fig. S4A and S4B]. First, focusing on endometrial and ovarian cancer, we examined levels of PARP1 and nucleolar DDX21 by IHC staining of commercial tissue microarrays. We found a significant association, wherein higher PARP1 correlated with a more prominent nucleolar localization of DDX21 across different types of cancers (Fig. 4A and B; Supplementary Fig. S4C). On the basis of this observation, we were interested in examining the potential of DDX21 as a prognostic biomarker. Thus, we first performed survival analysis from TCGA data, stratifying high-grade ovarian cancer and (uterine) endometrial cancer into high versus low DDX21 expression (RNA sequencing). A difference was only found to be statistically significant in endometrial cancer, where patients with high expression of DDX21 had poorer overal survival (Supplementary Fig. S4D). Based on this result, second, we evaluated DDX21 localization by IHC in our own cohort of 39 advanced stage endometrioid endometrial cancer patient samples. Advanced endometrial cancer is defined as stage III or stage IV disease at the time of presentation. These patients were chosen due to their higher risk of developing recurrent disease. Demographics, treatments, and outcomes of these patients are shown in Table 2 (Supplementary Table S2). Expression of nucleolar DDX21 was graded 0–2 and patients classified as low (0–1) or high (2) based on DDX21 IHC staining (Fig. 4C). Interestingly, high nucleolar localization of DDX21 was significantly associated with a decreased overall survival (OS, *P* = 0.0155) and progression-free survival (PFS, *P* = 0.0092; Fig. 4D and E). We further stratified into low grade (*n* = 27) versus high grade (*n* = 12) to examine nucleolar expression. While not statistically significant, there are more samples scoring lower in DDX21 nucleolar intensity in the lower grade group compared with high grade (Supplementary Fig. S4E). Excitingly, we also stratified the samples based on stage [stage IIIA (*n* = 5), IIIB (*n* = 2), IIIC1 (*n* = 11), IIIC2 (*n* = 12), and IVB (*n* = 9)]. DDX21 nucleolar localization was significantly different among different groups with higher DDX21 scoring in more advanced stages of endometrial cancer (*P* = 0.0041; Supplementary Fig. S4F). Our data suggest that DDX21 nucleolar localization (not necessarily overall expression level) associates with decreased survival and can potentially be used as a prognostic factor in patients with endometrial cancer.

## Discussion

We have previously uncovered a pathway for PARP1-dependent regulation of ribosome biogenesis and growth of breast cancer cells mediated through ADPRylation of DDX21. Herein, we have extended the observation of this mechanism in endometrial and ovarian cancers. We find that niraparib effectively disrupts the nucleolar localization of DDX21, which in turn prevents ribosome biogenesis, decreasing protein translation and ultimately negatively impacting endometrial and ovarian cancer cell growth. Importantly, we demonstrate (i) a significant negative correlation between nucleolar DDX21 intensity and PARPi sensitivity (high DDX21 = low IC_50_; low DDX21 = high IC_50_), and (ii) a significant association between high nucleolar DDX21 and decreased survival in patients with endometrial cancer. This strongly bolsters our hypothesis that nucleolar localization of DDX21 (not overall expression level) can predict response to PARPi. Collectively, our studies indicate that this pathway is active across multiple cancer types and can provide an alternative target for therapeutic action of PARPi.

### Translation Inhibition as a Therapeutic Target

Several studies have demonstrated that tumors require protein synthesis (translation) for growth and proliferation (39, 40). Many tumor suppressor genes and proto-oncogenes are involved in cancer development and progression by regulating ribosome biogenesis and protein translation (39, 40). Targeting the translation machinery is an established therapeutic strategy in cancers (39, 40). Inhibitors of PARP1 have been shown to improve clinical outcomes in several cancers, irrespective of *BRCA1/2* status. We have demonstrated that PARPi-positive therapeutic action is due to PARP1’s varied roles in many biological processes beyond DNA repair, such as its role in ribosome biogenesis and translation (through ADPRylation of DDX21). In future studies, it would be interesting to test the hypothesis that co-treatment with drugs inhibiting translation might potentiate the PARPi effect.

### Nucleolar DDX21 as a Prognostic Biomarker

In our study, we found an association between DDX21 nucleolar localization and elevated PARP1 levels using endometrial and ovarian cancer tissue microarrays (including pancreatic and prostate cancers). As proposed previously, we assume that PARP1-mediated ADPRylation of DDX21 is responsible for its proper localization and retention in nucleolus. However, our previous studies have demonstrated that while levels and patterns of ADPRylation can segregate ovarian cancers into distinct molecular phenotypes that can predict clinical outcome, the expression level of PARP1 itself is not a direct measure of its enzymatic activity (ADPRylation in the cell; ref. 41). Therefore, we suggest direct analysis of DDX21 localization might be a better predictor of clinical outcome than PARP1 levels. In our cell-based studies, cell lines with high nucleolar DDX21 are more sensitive to niraparib (lower IC_50_). In our patient cohort analysis, high levels of nucleolar DDX21, as measured by IHC, significantly associate with poorer survival for patients with endometrial cancer suggesting DDX21 to be a potential biomarker that can predict clinical outcomes. Further studies are warranted to see whether this association also exists in ovarian cancer.

### Localization of DDX21: A Biomarker for PARPi Response

Thus far, germline mutations in *BRCA1/2* (or other general defects in HR) have been used as predictors of PARP1 inhibitor efficacy due to the underlying synthetic lethal relationship between these. However, a recent study has comprehensively shown that in cell lines, HR status does not associate with sensitivity to PARPi, suggesting potential limitations in drug response data generated from cell lines (42). Another interpretation is that mechanisms beyond HR deficiency are also impacting response to PARPi. Consequently, the discovery of predictive biomarkers for PARPi sensitivity outside the setting of DNA damage is much needed. Herein, we propose DDX21 nucleolar localization as a novel biomarker that can predict clinical outcomes in patients treated with PARPi, independent of DNA damage. Because of the significant association between (i) PARP1 levels and DDX21 nucleolar localization, (ii) nucleolar DDX21 intensity and PARPi sensitivity (calculated IC_50_), and (iii) high nucleolar DDX21 associating with poorer survival, we hypothesize that high nucleolar DDX21 levels might provide useful information and predict whether a patient will benefit from treatment with PARPi. Testing this hypothesis will require getting access to gynecologic cancer patient data with matched pre- and post-PARPi treatment and following up on their clinical outcomes in relation to DDX21 localization.

In conclusion, our findings suggest that the use of PARPi can potentially be expanded to a broader population of patients with gynecologic cancers including those with *BRCA1/2* wild-type ovarian and endometrial cancers. Given the lack of reliable biomarkers for PARPi response outside of HR defects, here we present a unique potential biomarker, with the added advantage that we understand the molecular mechanism by which DDX21 nucleolar localization can predict response to PARPi. DDX21 nucleolar localization was associated with high PARP1 expression, higher sensitivity to PARPi, and decreased survival, warranting further research to validate that it can be used as a prognostic factor and as a biomarker of clinical response to PARPi.

## Supplementary Material

Supplementary Figure S1 Endometrial and ovarian cancer cell lines are HR-proficient

Supplementary Figure S2 PARP1 inhibition prevents ribosome biogenesis by inhibiting rRNA subunit production

Supplementary Figure S3 Niraparib decreases cell growth in endometrial and ovarian cancer cell lines

Supplementary Figure S4 PARP1 and DDX21 are more highly expressed in tumor tissues compared to normal tissues in different cancer types

Supplementary Table S1 Catalogue of publishes alterations in major genes involved in DNA damage repair

Supplementary Table S2 Demographic data of 39 advanced stage endometrioid endometrial cancer patients
