# Supplementary material for: Nucleolar Localization of the RNA Helicase DDX21 Predicts Survival Outcomes in Gynecologic Cancers
Source: Cancer Res Commun. 2024 Jun 13;4(6):1495–504. doi: 10.1158/2767-9764.CRC-24-0001 (PMC11172406; doi:10.1158/2767-9764.CRC-24-0001)
Supplement: Supplementary Figure S1 — Endometrial and ovarian cancer cell lines are HR-proficient [file crc-24-0001-s01.pdf]

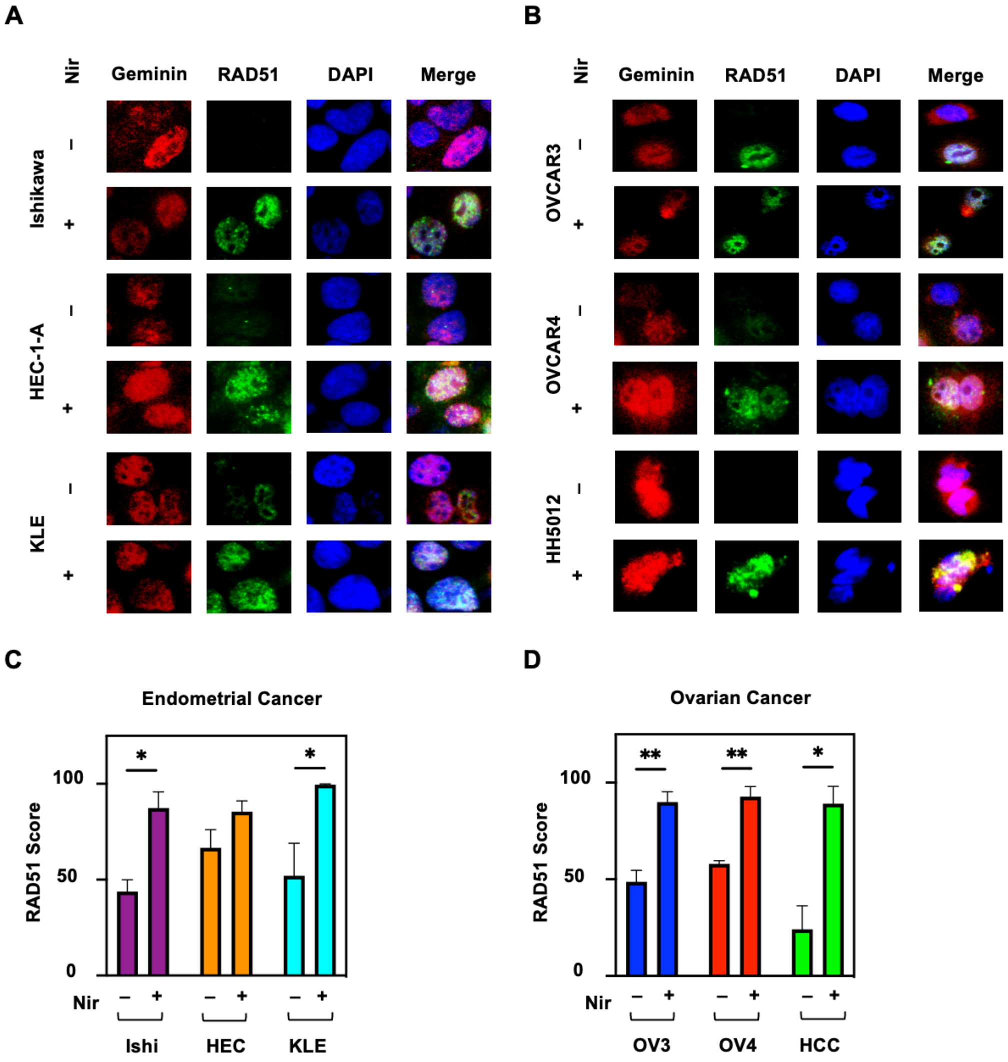

Figure S1. Related to Figure 1. Endometrial and ovarian cancer cell lines are HR-proficient.

(A and B) Endometroid endometrial cancer cell lines (Ishikawa, HEC-1-A, KLE) (A) and ovarian cancer cell lines (OVCAR3, OVCAR4, HCC5012) (B) can efficiently form RAD51 foci upon treatment with Niraparib, as measured by immunofluorescence.

(C and D) Quantification of the results from experiments shown in panels A and B showing the proportion of cells that are RAD51 positive out of a population of cells that are Geminin positive in endometrial cancer cell lines (C) and ovarian cancer cell lines (D). Each bar represents the mean + SEM; n = 3 (endometrial), n = 3 (ovarian). Bars marked with asterisks are significantly different; Student's t-test; * = p<0.05, ** = p<0.01.
