# Supplementary material for: Nucleolar Localization of the RNA Helicase DDX21 Predicts Survival Outcomes in Gynecologic Cancers
Source: Cancer Res Commun. 2024 Jun 13;4(6):1495–504. doi: 10.1158/2767-9764.CRC-24-0001 (PMC11172406; doi:10.1158/2767-9764.CRC-24-0001)
Supplement: Supplementary Figure S2 — PARP1 inhibition prevents ribosome biogenesis by inhibiting rRNA subunit production [file crc-24-0001-s02.pdf]

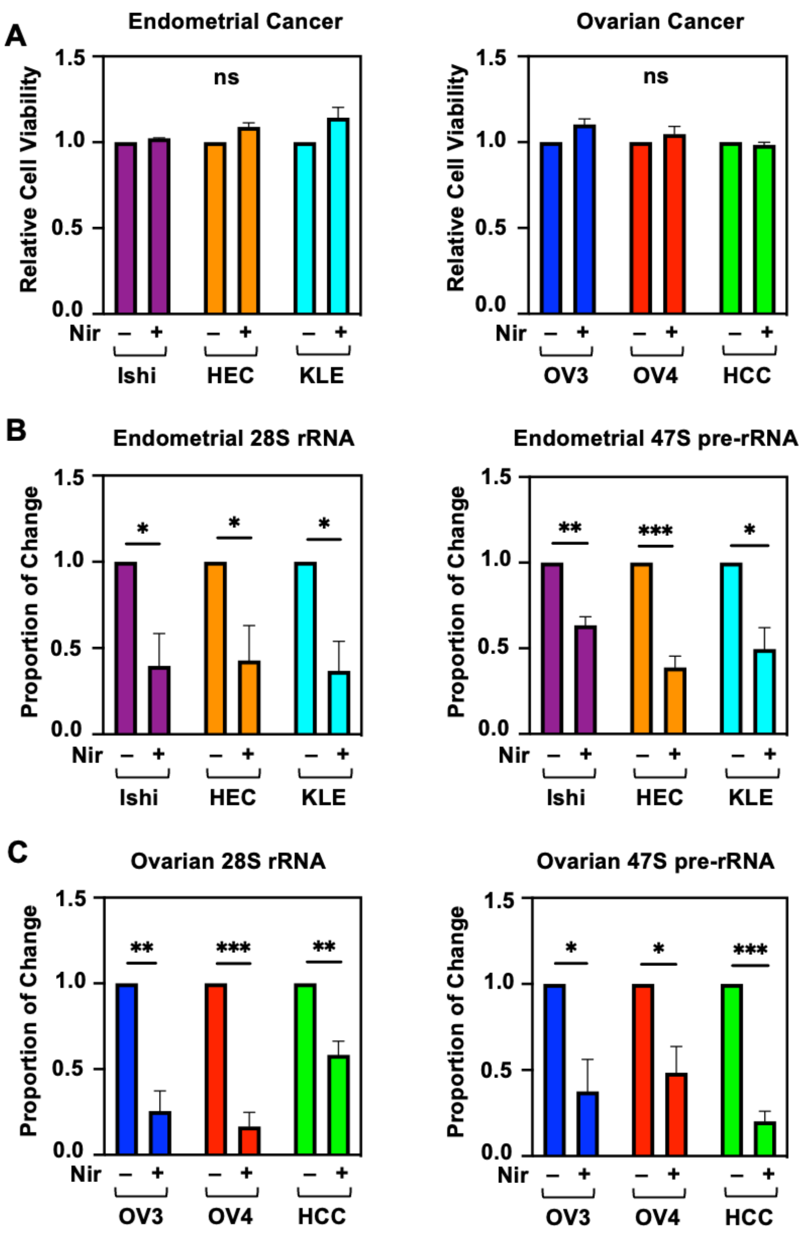

Figure S2. Related to Figure 2. PARP-1 inhibition prevents ribosome biogenesis by inhibiting rRNA subunit production.

(A) Treatment with 20 μM niraparib for 2 hours does not affect cell viability as measured by luminescence using CellTiter Glo assay in endometrial cancer cell lines (*left*) and ovarian cancer cell lines (*right*).

(B and C) Treatment with niraparib significantly decreases rRNA subunit (28S rRNA and 47S pre-rRNA) production in endometrial (B) and ovarian (C) cancer cells as assayed by qPCR. Each bar represents the mean + SEM; n=3. Bars marked with asterisks are significantly different; Student's t-test; * = $p<0.05$, ** = $p<0.01$, *** = $p<0.001$.
