# Supplementary material for: Nucleolar Localization of the RNA Helicase DDX21 Predicts Survival Outcomes in Gynecologic Cancers
Source: Cancer Res Commun. 2024 Jun 13;4(6):1495–504. doi: 10.1158/2767-9764.CRC-24-0001 (PMC11172406; doi:10.1158/2767-9764.CRC-24-0001)
Supplement: Supplementary Figure S3 — Niraparib decreases cell growth in endometrial and ovarian cancer cell lines [file crc-24-0001-s03.pdf]

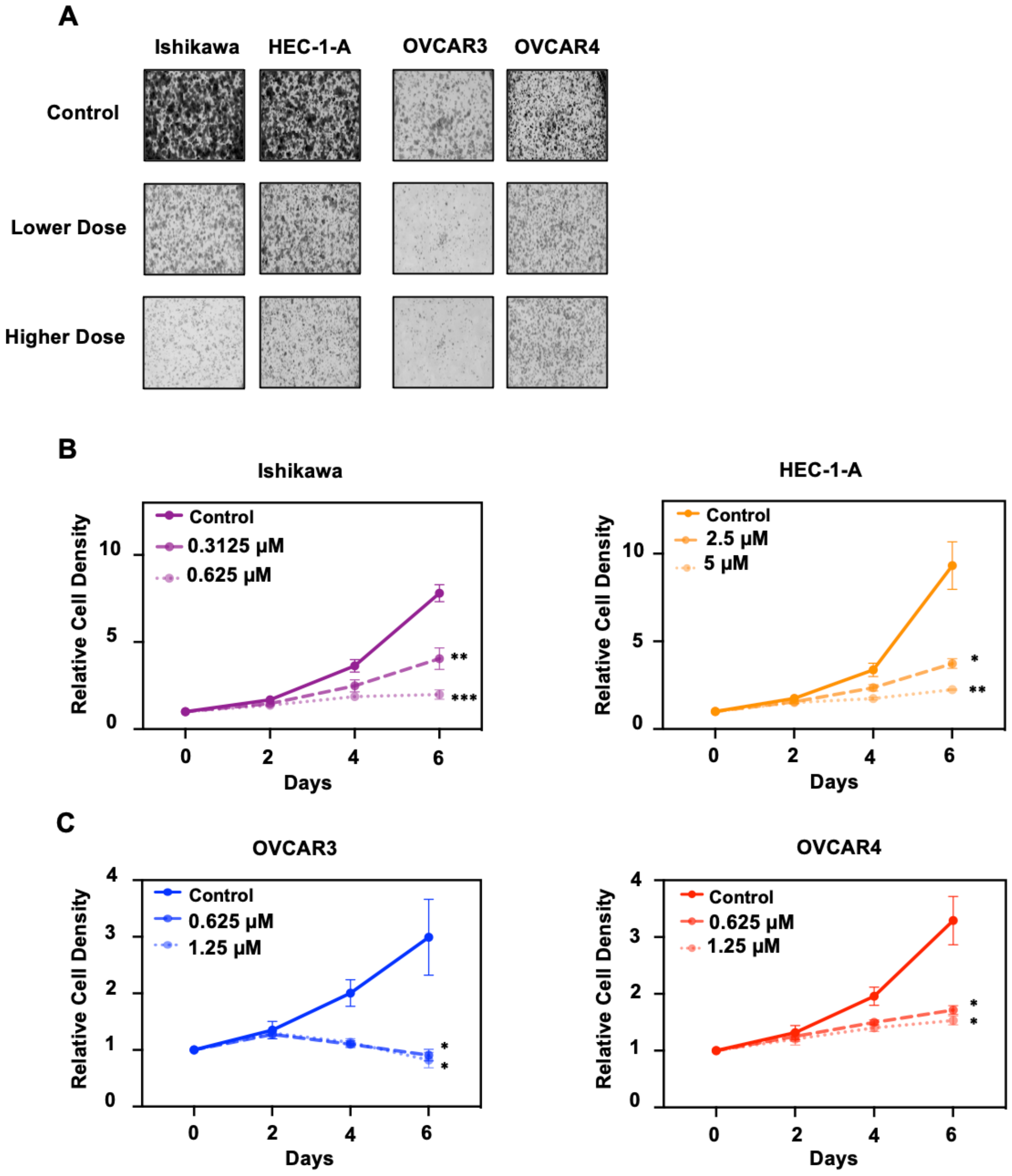

Figure S3. Related to Figure 3. Niraparib decreases cell growth in endometrial and ovarian and cancer cell lines.

(A) Representative images of crystal violet cell proliferation assay for endometrial and ovarian cancer cells with or without niraparib at 2 different doses (Dose 2 > Dose 1). For each cell line, dose 1 was chosen to be close to the IC50 and dose 2 was double dose 1.

(B and C) PARP1 inhibition with niraparib significantly decreases the proliferation of endometrial (B) and ovarian (C) cancer cell lines. Line graphs showing the growth of cells over a period of 6 days with different concentrations of niraparib. Each point represents the mean ± SEM; n=3. Points marked with asterisks are significantly different; Student's t-test; * = $p<0.05$, ** = $p<0.01$, *** = $p<0.001$.
