# Supplementary material for: Nucleolar Localization of the RNA Helicase DDX21 Predicts Survival Outcomes in Gynecologic Cancers
Source: Cancer Res Commun. 2024 Jun 13;4(6):1495–504. doi: 10.1158/2767-9764.CRC-24-0001 (PMC11172406; doi:10.1158/2767-9764.CRC-24-0001)
Supplement: Supplementary Figure S4 — PARP1 and DDX21 are more highly expressed in tumor tissues compared to normal tissues in different cancer types [file crc-24-0001-s04.pdf]

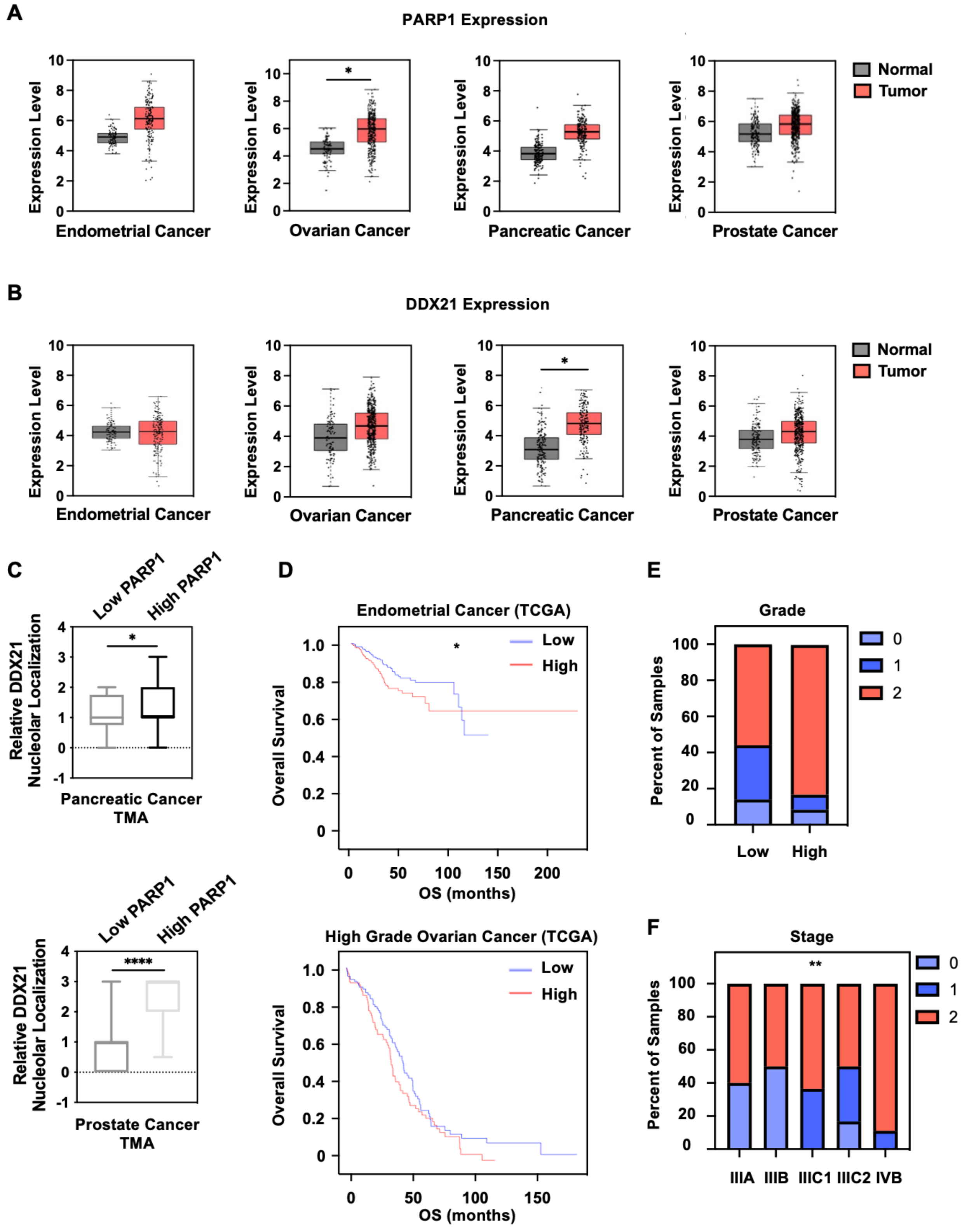
A
PARP1 Expression
Expression Level
Endometrial Cancer
Ovarian Cancer
Pancreatic Cancer
Prostate Cancer
Normal
Tumor
B
DDX21 Expression
C
Low PARP1
High PARP1
Relative DDX21 Nucleolar Localization
Pancreatic Cancer TMA
Prostate Cancer TMA
D
Endometrial Cancer (TCGA)
High Grade Ovarian Cancer (TCGA)
Overall Survival
OS (months)
Low
High
E
Grade
Percent of Samples
F
Stage
IIIA IIIB IIIC1 IIIC2 IVB

Figure S4. Related to Figure 4. PARP1 and DDX21 are more highly expressed in tumor tissues compared to normal tissues in different cancer types.

(A and B) RNA-seq expression data for PARP1 (A) and DDX21 (B) in endometrial (n = 174), ovarian (n = 426), pancreatic (n = 179), and prostate (n = 492) tumors (TCGA) compared to normal uterus (n = 91), ovary (n = 88), pancreas (n = 171) and prostate (n = 152) tissues (TCGA or GTEx). Bars marked with asterisks are significantly different; two-tailed Student's t-test; * = p<0.05)

(C) Box plots showing quantification of nucleolar DDX21 intensity in low- versus high-PARP1 pancreatic (*left panel)* and prostate cancer samples (*right panel*). Bars marked with asterisks are significantly different; Student's t-test; * = p<0.05, **** = p<0.0001.

(D) Survival analyses of (uterine) endometrial (*top*) and high-grade ovarian cancer patients (*bottom*), stratified by low versus high DDX21 (TCGA; RNA-Seq). ** p<0.01 for endometrial cancer patients; p<0.1 for high-grade ovarian cancer patients.

(E) For endometrial cancer, bar plot shows DDX21 nucleolar intensity stratified by low (grades 1-2; n=27) versus high (grade 3; n=12) grade. Kruskal-Wallis test; no significance.

(F) For endometrial cancer, bar plot shows DDX21 nucleolar intensity stratified by different stages; stage IIIA (n=5), IIIB (n=2), IIIC1 (n=11), IIIC2 (n=12) and IVB (n=9). Kruskal-Wallis test; ** p<0.01.
