# Supplementary material for: Nucleolar Localization of the RNA Helicase DDX21 Predicts Survival Outcomes in Gynecologic Cancers
Source: Cancer Res Commun. 2024 Jun 13;4(6):1495–504. doi: 10.1158/2767-9764.CRC-24-0001 (PMC11172406; doi:10.1158/2767-9764.CRC-24-0001)
Supplement: Supplementary Table S1 — Catalogue of publishes alterations in major genes involved in DNA damage repair [file crc-24-0001-s05.pdf]

Table S1. Related to Figure 1. Catalogue of published alterations in major genes involved in DNA damage repair.

	Ishikawa	HEC-1-A	KLE	OVCAR3	OVCAR4	HCC5012
ARID1A	Truncating mutation (putative driver) F2141Sfs*59; A339Lfs*24 [1] Truncating mutation (putative driver) F2141Sfs*59 [2]	Truncating mutation (putative driver) Q1835*; Q2115*; Q404H; G1761C [1, 2]	NA	Hom del [1]	NA	NA
ATM	Truncating mutation (putative driver) E1313Dfs*7 [1, 2]	Missense Mutation (putative passenger) P2353H [1, 2]	Truncating mutation (putative driver) S1905Ifs*25 [1]	Hom del [1]	Missense Mutation (putative passenger) N230T [1-3]	NA
BRCA1	Splice_rec, X1803_splice [1, 2] Missense Mutation P871L, S1634G [4]	NA	NA	NA	NA	NA
BRCA2	Missense Mutation N289H [4]	NA	Hom del [1, 2] Frame shift mutation A2852fs [4]	Hom del [1]	Missense Mutation (putative passenger) P2505L [1]	NA
CHEK2	NA	NA	NA	NA	NA	NA
PTEN	Truncating mutation (putative driver) T319*; V290* [1, 2]	NA	NA	NA	Amplification [1, 2]	NA
RAD51	NA	NA	NA	Hom del in RAD51D [1]	NA	NA
TP53	Missense Mutation (putative driver) M246V; D49H [1, 2]	Missense Mutation (putative driver) R248Q [1, 2]	Missense Mutation (putative driver) R175H [1, 2]	hom del [1] Missense Mutation (putative driver) R248Q [1, 2]	Missense Mutation (putative driver) L130V [1, 2]	TP53 mutation [5]

1. Ghandi, M., et al., *Next-generation characterization of the cancer cell line encyclopedia.* Nature, 2019. **569**(7757): p. 503-508.
2. Barretina, J., et al., *The Cancer Cell Line Encyclopedia enables predictive modelling of anticancer drug sensitivity.* Nature, 2012. **483**(7391): p. 603-607.
3. Reinhold, W.C., et al., *CellMiner: a web-based suite of genomic and pharmacologic tools to explore transcript and drug patterns in the NCI-60 cell line set.* Cancer research, 2012. **72**(14): p. 3499-3511.
4. Devor, E.J., et al., *Genomic characterization of five commonly used endometrial cancer cell lines.* International journal of oncology, 2020. **57**(6): p. 1348-1357.
5. Thu, K.L., et al., *A comprehensively characterized cell line panel highly representative of clinical ovarian high-grade serous carcinomas.* Oncotarget, 2017. **8**(31): p. 50489.

NA No evidence of alteration found in published sources.
