# Supplementary material for: Nucleolar Localization of the RNA Helicase DDX21 Predicts Survival Outcomes in Gynecologic Cancers
Source: Cancer Res Commun. 2024 Jun 13;4(6):1495–504. doi: 10.1158/2767-9764.CRC-24-0001 (PMC11172406; doi:10.1158/2767-9764.CRC-24-0001)
Supplement: Supplementary Table S2 — Demographic data of 39 advanced stage endometrioid endometrial cancer patients [file crc-24-0001-s06.pdf]

Table S2. Related to Figure 4. Demographic data of 39 advanced stage (stage III-IV) endometrioid endometrial cancer patients with mean age, percentage of patients in each category of race, stage, and grade, as well as mean progression free and overall survival. Data is expressed as mean or number of patients (percentage of total). Percentage may not add up to 100 due to rounding.

Demographic Data of Endometrial Cancer Patients (n = 39)

Variable	
Mean age at diagnosis (years)	53.7
Race	Number (%)
Caucasian	16 (41)
African American	6 (15.4)
Hispanic	2 (5.1)
Asian/other	15 (38.5)
Stage	Number (%)
IIIA	5 (12.8)
IIIB	2 (5.1)
IIIC1	11 (28.2)
IIIC2	12 (30.8)
IVA	0 (0)
IVB	9 (23.1)
Grade	Number (%)
1	13 (33.3)
2	14 (35.9)
3	12 (30.8)
Treatment	Number (%)
Neoadjuvant therapy	None
Primary treatment Surgery	39 (100) (samples collected at time of surgery)
Adjuvant therapy	31 (80)
Survival	Mean (months)
Progression-free survival	26.7
Overall survival	31.6
